# Association of exposure to air pollutants with gestational diabetes mellitus in Chiayi City, Taiwan

**DOI:** 10.3389/fendo.2022.1097270

**Published:** 2023-01-16

**Authors:** Yuan-Horng Yan, Chu-Chun Chien, Panchalli Wang, Mei-Chun Lu, Yu-Ching Wei, Jyh-Seng Wang, Jun-Sing Wang

**Affiliations:** ^1^ Department of Medical Research, Kuang Tien General Hospital, Taichung, Taiwan; ^2^ Department of Endocrinology and Metabolism, Kuang Tien General Hospital, Taichung, Taiwan; ^3^ Department of Nutrition and Institute of Biomedical Nutrition, Hung Kuang University, Taichung, Taiwan; ^4^ Department of Pathology, School of Medicine, College of Medicine, Kaohsiung Medical University, Kaohsiung, Taiwan; ^5^ Department of Pathology, Kaohsiung Municipal Ta-Tung Hospital, Kaohsiung, Taiwan; ^6^ Department of Obstetrics and Gynecology, Ditmanson Medical Foundation Chia-Yi Christian Hospital, Chiayi, Taiwan; ^7^ Department of Pathology and Laboratory Medicine, Kaohsiung Veterans General Hospital, Kaohsiung, Taiwan; ^8^ Division of Endocrinology and Metabolism, Department of Internal Medicine, Taichung Veterans General Hospital, Taichung, Taiwan; ^9^ Department of Medicine, School of Medicine, National Yang Ming Chiao Tung University, Taipei, Taiwan; ^10^ National Chung Hsing University, Taichung, Taiwan; ^11^ Department of Post-Baccalaureate Medicine, College of Medicine, National Chung Hsing University, Taichung, Taiwan

**Keywords:** air pollution, gestational diabetes mellitus, hyperglycemia, particulate matter, pregnancy

## Abstract

**Introduction:**

We investigated the associations of exposure to particulate matter with an aerodynamic diameter less than 2.5 μm (PM_2.5_) and several gaseous pollutants with risk of gestational diabetes mellitus (GDM) in Taiwan.

**Methods:**

We retrospectively identified pregnant women who underwent a two-step approach to screen for GDM between 2006 and 2014. Information on concentrations of air pollutants (including PM_2.5_, sulfur dioxide [SO_2_], nitrogen oxides [NO_x_], and ozone [O_3_]) were collected from a single fixed-site monitoring station. We conducted logistic regression analyses to determine the associations between exposure to air pollutants and risk of GDM.

**Results:**

A total of 11210 women were analyzed, and 705 were diagnosed with GDM. Exposure to PM_2.5_ during the second trimester was associated with a nearly 50% higher risk of GDM (odds ratio [OR] 1.47, 95% CI 0.96 to 2.24, p=0.077). The associations were consistent in the two-pollutant model (PM_2.5_ + SO_2_ [OR 1.73, p=0.038], PM_2.5_ + NO_x_ [OR 1.52, p=0.064], PM_2.5_ + O_3_ [OR 1.96, p=0.015]), and were more prominent in women with age <30 years and body mass index <25 kg/m^2^ (interaction p values <0.01).

**Discussion:**

Exposure to PM_2.5_ was associated with risk of GDM, especially in women who were younger or had a normal body mass index.

## Introduction

Air pollution is a serious global problem that increases disease burden and shortens life expectancy, especially in low- and middle-income countries (LMIC) (1–3). Global mortality attributable to exposure to particulate matter with an aerodynamic diameter less than 2.5 μm (PM_2.5_) increased by 20% from 1990 to 2015, accounting for 7.6% of total deaths in 2015 (2). Moreover, exposure to air pollutants has been associated with subclinical inflammation and insulin resistance (4). This may explain the association between exposure to PM_2.5_ and risk of incident diabetes (5).

As air pollution is a critical environmental issue in east and south Asia (2, 6), the number of people with incident diabetes and hyperglycemia in pregnancy is continuously increasing in these regions (7–10). The prevalence of diabetes in people aged 20-79 years significantly increased from 7.15% in 2005 to 10.10% in 2014 (p<0.001) in Taiwan (10). The numbers for incidence of diabetes were 11.9 per 10000 persons and 14.5 per 10000 persons (p<0.001), respectively (10). Similarly, the prevalence of GDM in Taiwan significantly increased from 7.6% in 2004 to 13.4% in 2015 (9). Physiologic insulin resistance could be noted in normal pregnancy, and gestational diabetes mellitus (GDM) may develop due to inadequate adaptation of insulin secretion (11, 12). In this context, GDM is often diagnosed in women with risk factors of abnormal glucose regulation.

The Diabetes Association of the Republic of China (DAROC) (http://www.endo-dm.org.tw/dia/) recommends universal screening for GDM for pregnant women with no history of diabetes at gestational week 24-28 using either one-step or two-step approach (13–15). Since chronic exposure to air pollutants may induce insulin resistance (4), exposure to PM_2.5_ has been associated with risk of GDM in a number of studies (16–18). However, there are inconsistent findings (19, 20) and data from east and south Asian countries are limited (13, 21). The Chiayi City is a mid-sized city located on the plains of southwestern Taiwan at a height of 69 m above sea level. Concentrations of air pollutants collected from a fixed-site monitoring station in this city could be representative of the residents’ ambient exposure to air pollutants. In this study, we aimed to investigate the associations of exposures to PM_2.5_ and several gaseous pollutants with risk of GDM in a group of pregnant women who underwent standard screening procedures in a hospital in the Chiayi City in Taiwan.

## Materials and methods

This study was conducted in accordance with the Declaration of Helsinki, and was approved (approval number: CYCH IRB No. 100006) by the Institutional Review Board of Ditmanson Medical Foundation Chia-Yi Christian Hospital. Informed consent was waived due to the retrospective study design and de-identification of data used in the analyses. We retrospectively identified pregnant women with no history of diabetes who underwent a two-step approach to screen for GDM (14, 15) at the Department of Obstetrics and Gynecology in the Ditmanson Medical Foundation Chia-Yi Christian Hospital between 2006 and 2014. Information on nulliparous/parous and body mass index (BMI) was collected from the electronic medical records.

**Figure 1:**
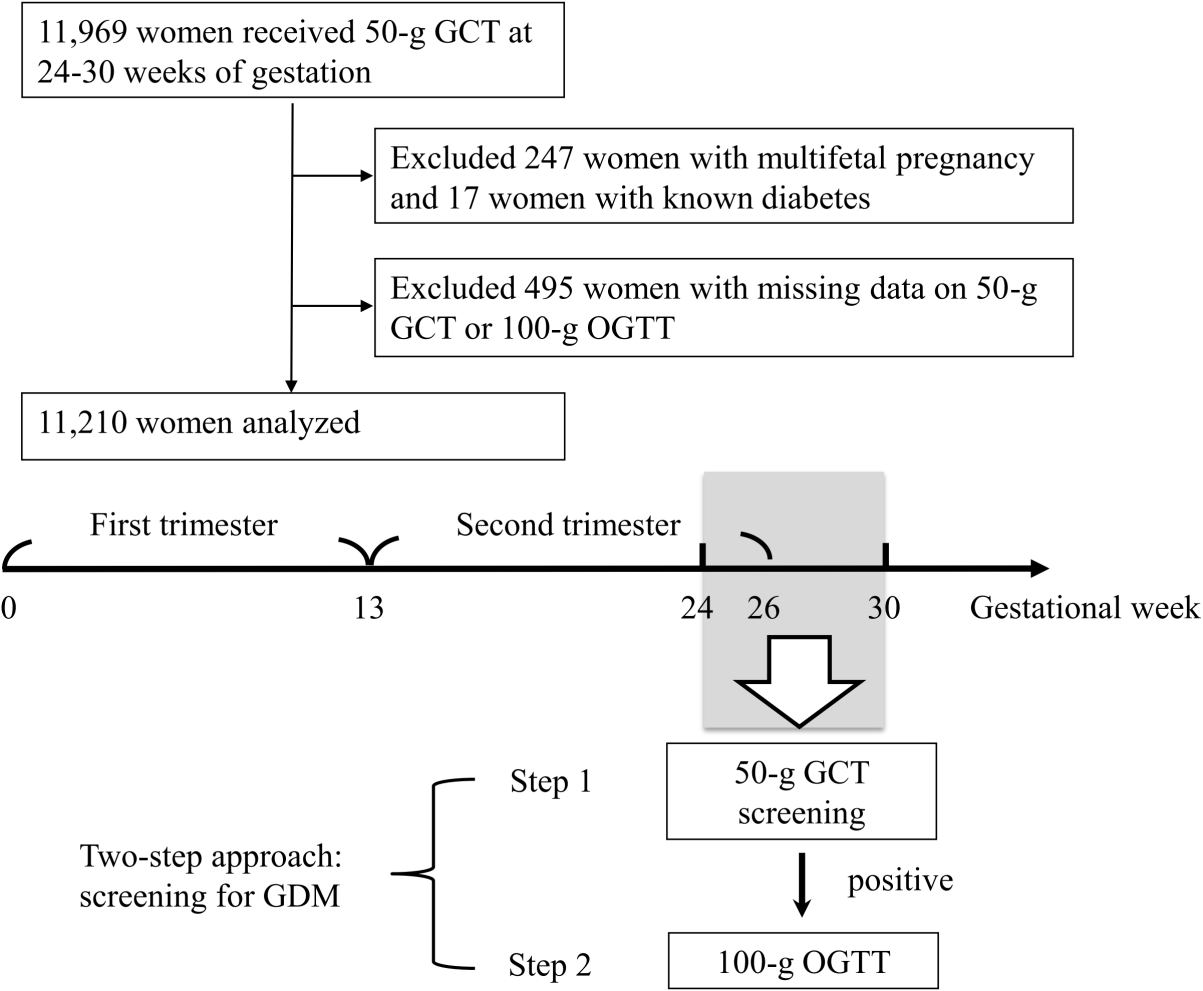
Enrollment of the study population. GCT, glucose challenge test. GDM, gestational diabetes mellitus. OGTT, oral glucose tolerance test.

All women underwent a 50-g glucose challenge test (GCT) at 24-28 weeks of gestation (**Figure 1**) (14, 15, 22). If the 1-hour plasma glucose after the 50-g GCT was ≥ 140 mg/dl, the women undertook a 100-g oral glucose tolerance test (OGTT) (23) before 30 weeks of gestation. The 100-g OGTT was conducted in the morning after an overnight fast at an outpatient clinic. Plasma glucose levels were examined at 4 time points (fasting, and 1 hour, 2 hours, and 3 hours after the 100-g OGTT). GDM was diagnosed if there were two or more of the plasma glucose levels higher than the cutoff values (≥ 95, 180, 155, and 140 mg/dl, respectively) based on international criteria (14, 15, 22, 23). All venous plasma glucose levels were determined using the hexokinase-G6PDH method with a Hitachi 7170 automatic analyzer (Hitachi Co., Tokyo, Japan).

Information on concentrations of air pollutants was collected from a single fixed-site monitoring station (Chiayi City station), and the temporal distribution of exposure was used for analyses. All study participants were residents of Chiayi City. The city is located on the plains of southwestern Taiwan at a height of 69 m above sea level (https://twinfo.ncl.edu.tw/tiqry/hypage.cgi?HYPAGE=search/search_res.hpg&dtd_id=4&g=0&sysid=00000014). The east-west and north-south diameters of the city measure 15.8 and 10.5 km, respectively. Hence, the data from the Chiayi City station were considered representative of the residents’ ambient exposure to air pollutants. Concentrations of air pollutants (including PM_2.5_, sulfur dioxide [SO_2_], nitrogen oxides [NO_x_], and ozone [O_3_]) and meteorological measurements (temperature and humidity) were collected (https://airtw.epa.gov.tw/ENG/default.aspx). We calculated the daily average concentrations of air pollutants for analyses (24, 25). To examine the associations between exposure to air pollutants at various periods and risk of GDM, the mean concentrations of air pollutants during the first (1-13 gestational weeks) and the second (14-26 gestational weeks) trimester were determined. We also calculated different time windows of exposure to air pollutants (1-6 months) before the 50-g GCT.

All statistical analyses were performed using SAS 9.4 (SAS Institute, Cary, NC). A two-sided p value of <0.05 was considered statistically significant. We conducted logistic regression analyses to determine the associations between exposure to air pollutants (PM_2.5_, SO_2_, NO_x_, and O_3_) and risk of GDM. The models were adjusted for age, nulliparous/parous, BMI, season/year, and the moving averages of humidity and temperature. We also investigated the associations between exposure to two pollutants (PM_2.5_ + SO_2_, PM_2.5_ + NO_x_, and PM_2.5_ + O_3_) and risk of GDM. Last, the aforementioned associations were examined in various subgroups (age <30 vs. ≥ 30 years, nulliparous yes vs. no, BMI <25 vs. ≥ 25 kg/m^2^).

## Results

A total of 11210 women were analyzed (mean age 29.3 ± 4.8 years, mean BMI 24.8 ± 3.6 kg/m^2^, 72.9% nulliparous, **Table 1**). All women underwent a 50-g GCT at 24-28 weeks of gestation, and 2545 of them had a 1-hour plasma glucose ≥ 140 mg/dl. A 100-g OGTT was conducted for these women before 30 weeks of gestation, and 705 were diagnosed with GDM. The mean concentrations of air pollutants during the first trimester, the second trimester, and 1-6 months before the 50-g GCT are shown in **Table 2**.

**Table 1:** Characteristics of the study population.

Variables	Number of women	% or mean ± SD
Maternal age, years	11210	29.3 ± 4.8
Nulliparous		
Yes	8177	72.9
No	3033	27.1
BMI at 50-g GCT, kg/m^2^	10592	24.8 ± 3.6
Gestational week at 50-g GCT	11210	26.3 ± 1.5
Glucose regulation status		
GDM	705	6.3
Non-GDM	10505	93.7
GCT results		
50-g GCT, mg/dl	11210	122.7 ± 28.3
100-g GCT, mg/dl		
Fasting	2545	83.3 ± 10.4
1-hour	2545	161.9 ± 32.5
2-hour	2545	144.4 ± 32.8
3-hour	2545	116.2 ± 29.5

BMI, body mass index. GCT, glucose challenge test. GDM, gestational diabetes mellitus.

**Table 2:** Mean concentrations of air pollutants during a defined period.

Period of observation	PM_2.5_ (μg/m^3^)	SO_2_ (ppb)	NO_x_ (ppb)	O_3_ (ppb)
First trimester	43.32 ± 13.35	3.87 ± 0.82	20.30 ± 6.98	27.46 ± 4.14
Second trimester	42.30 ± 13.39	3.82 ± 0.81	19.61 ± 6.93	28.07 ± 4.12
Before 50-g GCT				
1 month	41.34 ± 15.76	3.76 ± 0.95	19.27 ± 7.64	28.01 ± 6.06
2 months	41.29 ± 14.56	3.76 ± 0.88	19.26 ± 7.28	28.01 ± 5.02
3 months	41.44 ± 13.41	3.76 ± 0.81	19.32 ± 6.84	27.96 ± 4.10
4 months	41.74 ± 12.19	3.78 ± 0.73	19.45 ± 6.31	27.88 ± 3.27
5 months	42.11 ± 10.95	3.80 ± 0.67	19.62 ± 5.71	27.81 ± 2.64
6 months	42.47 ± 9.70	3.82 ± 0.60	19.79 ± 5.04	27.73 ± 2.29

Data are mean ± SD. GCT, glucose challenge test. PM, particulate matter. SO_2_, sulfur dioxide. NO_x_, nitrogen oxides. O_3_, ozone.

**Table 3** shows the associations between mean concentrations of air pollutants during the first and second trimester and diagnosis of GDM. Exposure to air pollutants during the first trimester was not associated with risk of GDM. In contrast, exposure to PM_2.5_ during the second trimester was associated with a nearly 50% higher risk of GDM (odds ratio 1.47, 95% CI 0.96 to 2.24, p=0.077). The associations were consistent in the two-pollutant model (PM_2.5_ + SO_2_ [odds ratio 1.73, 95% CI 1.03 to 2.92, p=0.038], PM_2.5_ + NO_x_ [odds ratio 1.52, 95% CI 0.98 to 2.35, p=0.064], PM_2.5_ + O_3_ [odds ratio 1.96, 95% CI 1.14 to 3.35, p=0.015]).

**Table 3:** Associations between mean concentrations of air pollutants and GDM.

	First trimester	Second trimester
Single-pollutant model^a^		
PM_2.5_	0.96 (0.68-1.34)	1.47 (0.96-2.24)
SO_2_	0.90 (0.67-1.22)	1.03 (0.70-1.52)
NO_x_	1.07 (0.61-1.89)	1.00 (0.49-2.02)
O_3_	1.01 (0.87-1.17)	0.98 (0.81-1.17)
Two-pollutant model^a^		
PM_2.5_ + SO_2_	1.04 (0.68-1.58)	1.73 (1.03-2.92)^*^
PM_2.5_ + NO_x_	0.93 (0.64-1.35)	1.52 (0.98-2.35)
PM_2.5_ + O_3_	0.92 (0.61-1.39)	1.96 (1.14-3.35)^*^

Data are odds ratio (95% CI). GDM, gestational diabetes mellitus. PM, particulate matter. SO_2_, sulfur dioxide. NO_x_, nitrogen oxides. O_3_, ozone. ^a^Logistic regressions with adjustments for individual-specific effects (age, nulliparous/parous, body mass index, season, and year) and for moving averages of humidity and temperature in the corresponding study period. ^*^P<0.05.

**Figure 2:**
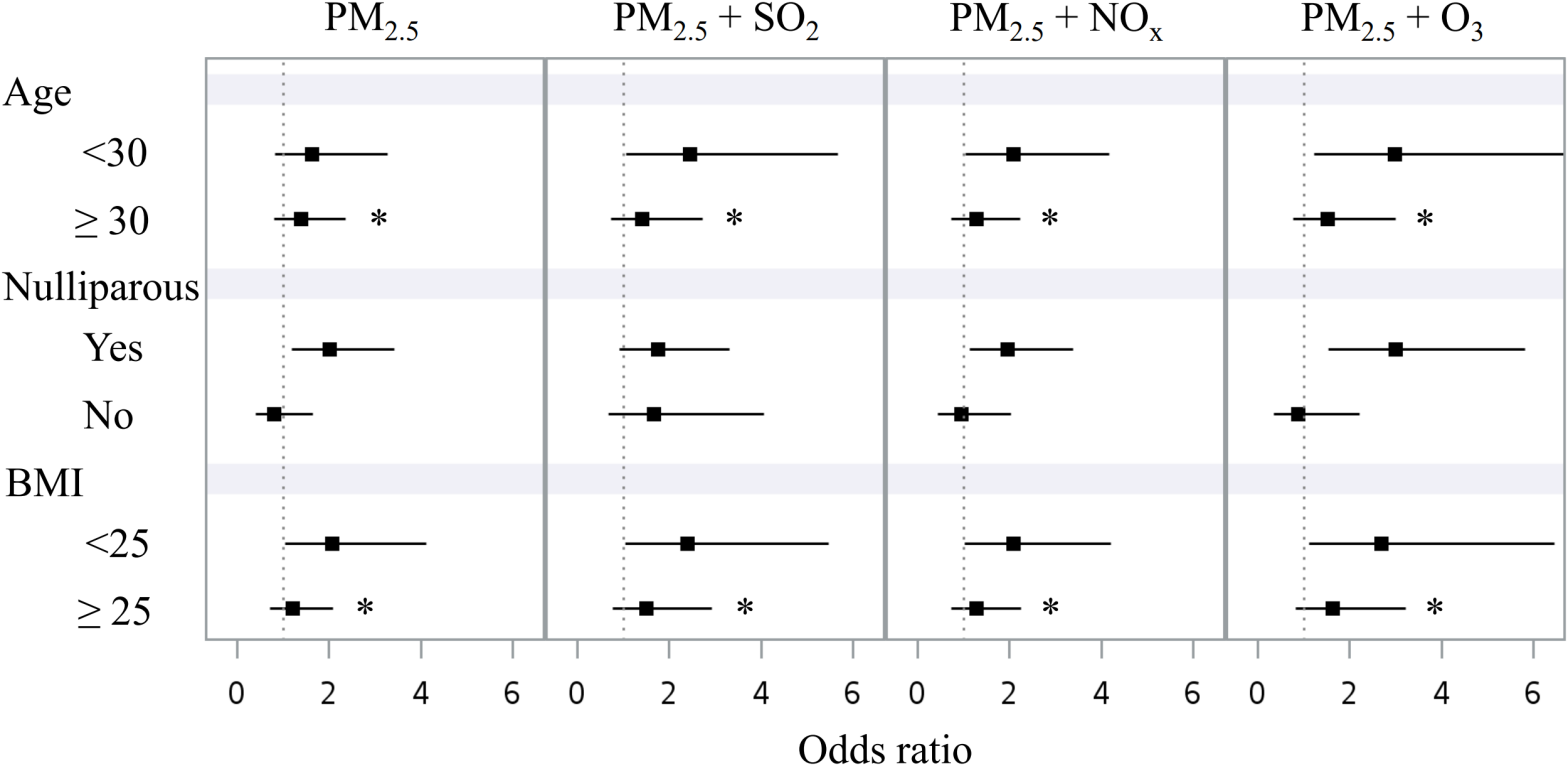
Forest plot displaying the associations between mean concentrations of air pollutants in the second trimester and GDM in different subgroups: age (<30 vs. ≥ 30 years), nulliparous (yes vs. no), and BMI (<25 vs. ≥ 25 kg/m^2^). BMI, body mass index. GDM, gestational diabetes mellitus. PM_2.5_, particulate matter with an aerodynamic diameter less than 2.5 μm. SO_2_, sulfur dioxide. NO_x_, nitrogen oxides. O_3_, ozone. *p interaction <0.01.

**Figure 2** shows the associations between exposure to air pollutants during the second trimester and GDM among various subgroups (age <30 vs. ≥ 30 years, nulliparous yes vs. no, BMI <25 vs. ≥ 25 kg/m^2^). The associations were more prominent in women with age <30 years, nulliparous, and BMI <25 kg/m^2^. Interaction p values were <0.01 for the age and BMI subgroups.

**Table 4** shows the associations between exposure to air pollutants 1-6 months before the 50-g GCT and GDM. There were no significant associations when exposure to PM_2.5_ for 1-3 months prior to the 50-g GCT was examined. However, we observed significant associations between exposure to PM_2.5_ for 4-6 months prior to the 50-g GCT and GDM. There were no significant associations between exposure to SO_2_, NO_x_, O_3_, and GDM in this model.

**Table 4:** Associations between mean concentrations of air pollutants during a defined period and GDM^a^.

	PM_2.5_	SO_2_	NO_x_	O_3_
Before 50-g GCT				
1 month	1.04 (0.78-1.39)	1.00 (0.77-1.29)	1.00 (0.67-1.51)	0.94 (0.78-1.13)
2 months	1.28 (0.93-1.76)	1.01 (0.78-1.31)	0.99 (0.62-1.58)	0.96 (0.81-1.13)
3 months	1.36 (0.97-1.90)	0.95 (0.71-1.28)	0.85 (0.49-1.45)	0.94 (0.81-1.09)
4 months	1.53 (1.04-2.26)^*^	0.90 (0.65-1.26)	0.70 (0.40-1.22)	0.97 (0.84-1.12)
5 months	1.81 (1.19-2.76)^*^	0.89 (0.63-1.25)	0.78 (0.44-1.39)	0.97 (0.84-1.11)
6 months	1.60 (1.06-2.41)^*^	0.87 (0.63-1.20)	0.86 (0.49-1.51)	0.98 (0.84-1.13)

Data are odds ratio (95% CI). GCT, glucose challenge test. GDM, gestational diabetes mellitus. PM, particulate matter. SO_2_, sulfur dioxide. NO_x_, nitrogen oxides. O_3_, ozone. ^a^Logistic regressions with adjustments for individual-specific effects (age, nulliparous/parous, body mass index, season, and year) and for moving averages of humidity and temperature in the corresponding study period. ^*^P<0.05.

## Discussion

In this study, we demonstrated that exposure to PM_2.5_ was associated with risk of GDM in women with no history of diabetes. The association was significant when considering the exposure in the second trimester (especially in the two-pollutant model, **Table 3**) or for 4-6 months before the 50-g GCT (**Table 4**). In the subgroup analyses, an association was observed in women who had an age <30 years or a BMI <25 kg/m^2^ (**Figure 2**). Our findings suggest that exposure to PM_2.5_ was associated with risk of GDM, especially in women who were younger or had a normal body mass index. These results may have implications for the healthcare of patients with GDM in regions with serious air pollution (2, 6) and a high prevalence of hyperglycemia in pregnancy (7–9).

Exposure to air pollutants has been associated with risk of GDM, although there are inconsistent findings. In a recent study (26), PM_2.5_ exposure during pregnancy was associated with a higher risk of GDM in a large US cohort. In contrast, no association was noted between PM_2.5_ exposure in the first or second trimester and risk of GDM in another US cohort (20). Differences in the methods used to diagnose GDM, investigations of various gaseous pollutants, and different covariates adjusted in the analytic models may partly explain the inconsistent results in previous studies (16–20, 26–29). All study participants were screened for GDM using the standard “two-step approach” (14, 15) in this study, and our findings are consistent with a recent meta-analysis (18) in which an association between the second trimester exposure to PM_2.5_ and risk of GDM was observed.

Air pollution is a serious issue in east and south Asia (2, 6) where the prevalence of GDM is high (7–9). Investigations on the associations between exposure to air pollutants and GDM in these regions are important, but relevant data are limited (13, 21). The level of PM_2.5_ in a study conducted in the US (20) was around 10-11 μg/m^3^. In a previous study in Taiwan (21) based on data from all 76 fixed-site air quality monitoring station, the level was around 30 μg/m^3^. The authors reported a significant association between PM_2.5_ exposure and risk of GDM. In this study, the mean level of PM_2.5_ was around 41-43 μg/m^3^ (**Table 2**). Using the same screening approach, the rate of GDM in the study conducted in the US (20) was 3.4% (vs. 6.3% in our cohort). We suggest that exposure to air pollutants may contribute to the high prevalence of abnormal glucose regulation during pregnancy in east and south Asia (7–9, 13, 21). Our findings were consistent with recent studies in China (30–32). In these studies, the median level of PM_2.5_ was around 70-80 μg/m^3^ (30, 31).

The significant associations between the two-pollutant models and GDM (**Table 3**) are not surprising, since the gaseous pollutants (SO_2_, NO_x_, and O_3_) have been associated with abnormal glucose regulation in pregnancy (33–39). It is interesting to note that the association was mainly observed in women with an age <30 years or a BMI <25 kg/m^2^ (**Figure 2**). Insulin resistance associated with weight gain during pregnancy may contribute to the development of abnormal glucose regulation (11, 12). Furthermore, exposure to PM_2.5_ has been shown to exaggerate insulin resistance in animal models (40, 41). This effect might be more prominent in people at a relatively low insulin resistance state (younger or leaner), which merits further investigations.

This study has several limitations. First, the causality of exposure to air pollutants and GDM cannot be confirmed with a retrospective study design. Second, there were considerable variations in the interactions between the environment and human subjects (42). The bias in the quantification of human exposure to air pollutants might have confounded our results. Nevertheless, monitoring individual exposure to air pollution in a large cohort is difficult in a real-world setting. Our cohort was selected from a single medical center and all of the study participants resided in Chiayi City (a mid-sized city located on the plains of southwestern Taiwan). In this context, using data from the Chiayi City station to estimate exposure to ambient air pollutants may have minimized the aforementioned bias. Final, we acknowledge that some factors may influence the effects of exposure to air pollutants on glucose regulation, such as dietary, genetic, and occupational factors (43–45). These factors were not examined in this study, and should be considered when interpreting our results.

## Conclusion

In summary, we demonstrated that exposure to PM_2.5_ was associated with risk of GDM, especially in women who were younger or had a normal body mass index. Our results are consistent with previous studies conducted in regions with high levels of PM_2.5_ (30–32). The differences in associations among subgroups require further investigations.

The aforementioned findings may have implications for the healthcare of patients with GDM in east and south Asia where the air pollution is serious. Universal screening for GDM in pregnant women with no history of diabetes may be required. National policy and efforts to improve air quality might help reduce the prevalence of abnormal glucose regulation in these regions.

## Data availability statement

The datasets presented in this article are not readily available because of privacy/ethical restrictions. Requests to access the datasets should be directed to Jun-SW, jswang@vghtc.gov.tw.

## Ethics statement

The studies involving human participants were reviewed and approved by the Institutional Review Board of Ditmanson Medical Foundation Chia-Yi Christian Hospital. Written informed consent for participation was not required for this study in accordance with the national legislation and the institutional requirements.

## Author contributions

Y-HY, C-CC, PW, and M-CL contributed to conception and design of the study. Y-HY, Y-CW, Jyh-SW, and Jun-SW organized the database. Y-HY and M-CL performed the statistical analysis. Y-HY, C-CC, and Jun-SW wrote the first draft of the manuscript. PW, M-CL, Y-CW, and Jyh-SW reviewed and edited the manuscript. All authors contributed to the article and approved the submitted version.
